# Echocardiographic Findings in Cardiomyopathy Due to Acromegaly

**DOI:** 10.3390/biomedicines13030605

**Published:** 2025-03-01

**Authors:** Oscar Orihuela Rodríguez, Leobardo Valle Nava, Aldo Ferreira-Hermosillo, Héctor A. Carmona-Ruiz, Ariana Acevedo Meléndez, Andrés Jacobo Ruvalcaba, Ernesto Sosa-Eroza

**Affiliations:** 1 Clinical Department of Cardiology, Hospital de Especialidades “Dr. Benardo Sepúlveda”, Centro Médico Nacional Siglo XXI, Instituto Mexicano del Seguro Social, Mexico City 06720, Mexico; leonavatkd2@gmail.com (L.V.N.); drhacr80@gmail.com (H.A.C.-R.); draarianaacevedo@gmail.com (A.A.M.); electrocardiologia2016@gmail.com (A.J.R.); 2 Unidad de Investigación Médica en Enfermedades Endocrinas, Centro Médico Nacional Siglo XXI, Instituto Mexicano del Seguro Social, Mexico City 06720, Mexico; aferreira@endocrinologia.org.mx; 3 Servicio de Endocrinología, Hospital de Especialidades “Dr. Benardo Sepúlveda”, Centro Médico Nacional Siglo XXI, Instituto Mexicano del Seguro Social, Mexico City 06720, Mexico; esosae@yahoo.com

**Keywords:** echocardiography, acromegaly, neuroendocrine tumors, cardiovascular diseases

## Abstract

**Background**: Cardiomyopathy is the leading cause of morbidity and mortality in patients with acromegaly. Pharmacological and surgical treatment of the disease has been associated with morphological and functional benefits for the heart, but other studies have shown that the condition and its effects may be irreversible. This study aims to uncover the most frequent echocardiographic changes in patients with cardiomyopathy due to acromegaly. **Methods**: An observational, descriptive, cross-sectional study was performed. Patients were referred from the Endocrinology department to the Cardiology department. This study was conducted from November 2020 to November 2022. Patients with the following criteria were included: over 18 years of age, of both genders, and with a complete clinical record and complete laboratory studies. **Results**: A total of 89 men (38%) and 148 women (62%) were included, with a mean age of 48 ± 12 years in the men and 49 ± 13 years in the women (*p* = 0.223). The most frequent cardiac findings were concentric hypertrophy (CHT) in 116 patients (49%), concentric remodeling (CR) in 52 patients (22%), and eccentric hypertrophy (EH) in 18 patients (8%). The left ventricular ejection fraction (LVEF) was preserved in the entire population. Left atrial enlargement (LAE) was observed in 88 patients (37%), diastolic dysfunction in 61 patients (26%), right ventricular dilatation in 47 patients (20%), right atrial enlargement in 120 patients (51), and pulmonary hypertension in 28 patients (12%). Valvular insufficiencies (VIs) were observed: tricuspid VIs in 73%, mitral VIs in 49%, and aortic VIs in 24% of the population. **Conclusions**: The frequency of changes in the four chambers is elevated in cardiomyopathy due to acromegaly.

## 1. Introduction

Acromegaly is a disease caused by excessive secretion of growth hormone (GH) and hepatic production of insulin-like growth factor type 1 (IGF-I), caused in 98% of cases by a GH-secreting pituitary adenoma [1]. It is a relatively rare condition, with a prevalence of 18 cases per million inhabitants in Mexico [2], where it is an underdiagnosed disease [3].

Active and untreated acromegaly is associated with cardiovascular morbidity and mortality in up to 80% of cases [4,5]. The development of cardiomyopathy due to acromegaly is divided into three stages depending on the time of evolution: early (<5 years), characterized by increased cardiac output and decreased peripheral vascular resistance; middle (>5 years), characterized by concentric hypertrophy and diastolic dysfunction; and late (>15 years), where congestive heart failure occurs [6]. Standard Doppler echocardiography is used to evaluate cardiac function and morphology, as it is non-invasive, high-resolution, of an accessible cost, and reproducible [7].

Using surgery, pharmacological treatment, radiotherapy, or a combination of these treatments, left ventricular hypertrophy (LVH), ventricular filling alterations, and myocardial damage decrease significantly [8]. However, cardiovascular changes become irreversible after a prolonged period of excess production of GH and IGF-1 [9]. Since acromegaly is often diagnosed late in its progression, related cardiac complications can contribute to death in up to 60% of patients at the time of diagnosis [10]. After 15 years of evolution without treatment, it reaches 100% mortality [11].

This study aimed to characterize the cardiovascular alterations diagnosed using echocardiography in a cohort of Mexican patients with acromegaly.

## 2. Materials and Methods

An observational, descriptive, cross-sectional study was conducted at the Unidad Médica de Alta Especialidad, Hospital de Especialidades del Centro Médico Nacional Siglo XXI del Instituto Mexicano del Seguro Social. The protocol was approved by the ethics and research committee (R: 2020-3601-227), and this study was conducted from November 2020 to November 2023. Patients were referred from the Endocrinology department to the Cardiology department. They included those who met the following criteria: having a diagnosis of acromegaly, being over 18 years of age, either gender, and having complete clinical records and laboratory studies. Patients with previously detected heart disease and those without complete biochemical studies were excluded. The protocol and aim of this study were fully explained to the subjects, who gave their written consent.

### 2.1. Echocardiograms

Echocardiographic studies were performed using commercially available echocardiography equipment (iE33^®^, Philips Medical System, Andover, MA, USA). Images were obtained in the 2-D and M modes from the parasternal and apical approaches with the patient in the left lateral decubitus position. Measurements were made following the recommendations of the American Society of Echocardiography [12].

The left ventricular (LV) mass was calculated using the Deveraux formula, and left ventricular hypertrophy was defined as a mass greater than 95 g/m^2^ in women and greater than 115 g/m^2^ in men. Body surface area was calculated using Mosteller’s formula [13]. LV diastolic function was assessed following the recommendations of the American Society of Echocardiography, published in 2016, which considered diastolic dysfunction when at least 3 of the following criteria were met: an average E/e′ ratio > 14, a septal e′ velocity < 7 cm/s, a lateral e′ velocity < 10 cm/s, a tricuspid regurgitation velocity > 2.8 m/s, or a left atrial indexed volume > 34 mL/m^2^ [14].

The modified Bernoulli equation was used to estimate pulmonary artery systolic pressure to obtain the gradient between the right atrial and left ventricular pressure. The value obtained was added to the right atrial pressure estimated using the diameter of the inferior vena cava. The present study defined pulmonary hypertension as a pulmonary artery systolic pressure (PSAP) >35 mmHg [15].

Hormonal laboratory studies were conducted using an electrochemiluminescence technique for free thyroxine (reference value (ref. value): 0.93–1.70 ng/dL), stimulating thyroid hormone (ref. value: 0.27–4.2 μIU/mL), and prolactin (ref. value: 4.1–18.4 ng/mL), with COBAS 6000 Roche Diagnostics equipment (Basel, Switzerland), and using a chemiluminescence technique for GH (ref. val. 0.02–4.77 ng/mL) and IGF-1, with LIAISON analyzer equipment (DiaSorin, Saluggia, Italy). The ref. value for IGF-1 is sex- and age-distributed, as shown in Table 1.

### 2.2. Statistical Analysis

Descriptive statistics were calculated for the demographic and echocardiographic variables. The distribution of the data was evaluated using the Kolmogorov–Smirnov test. Data are described as means and standard deviations or medians and interquartile ranges (IQRs) for quantitative variables and percentages for dichotomous variables.

The variables were analyzed according to sex to evaluate whether there was a difference among groups by applying Student’s *t*-test or the Mann–Whitney U test to quantitative variables and the chi-squared test to qualitative variables. We also evaluated the correlation of the echocardiographic variables with IGF-1 levels, as well as with the duration of the disease, using Spearman’s correlation test. The IBM SPSS Statistics statistical program version 25.0 was used. Values of *p* < 0.05 were considered significant.

## 3. Results

### 3.1. The Study Population

A total of 237 patients with acromegaly were evaluated, 89 men (38%) and 148 women (62%), with a mean age of 49 ±13 years, weight of 78 (68–88) kg, and BMI of 29.2 (26.0–33.7) kg/m^2^. The most frequently reported cardiovascular risk factors were obesity (45%), systemic arterial hypertension (35%), type 2 diabetes mellitus (36%), dyslipidemia (14%), and smoking (7%). Regarding comorbidities, obesity and hypothyroidism were more frequent in the women; meanwhile, secondary hypogonadism was most frequently reported in the men. Despite the weight being higher in the men, the BMI did not differ among the groups. No other differences were observed in the rest of the comorbidities (Table 2). We also observed that 11% of the patients (*n* = 26) had fasting glucose levels greater than 126 mg/dL, 31% had cholesterol levels greater than 200 mg/dL (*n* = 74), and 43% (*n* = 102) had triglyceride levels greater than 150 mg/dL. As expected, we found that the men had higher levels of hemoglobin, hematocrit, and creatinine. Meanwhile, the women had higher levels of glucose, total cholesterol, HDL-c, and triglycerides (Table 3).

The specific treatment for acromegaly was surgical, medical, and/or radiotherapeutic. Surgical treatment consisted of resection of the pituitary tumor in transsphenoidal surgery with the endoscopic technique in 40 (25%) patients. Medical treatment was administered to 62% (*n* = 147) of the patients and consisted of somatostatin analogs (lanreotide or octreotide), cabergoline, or a combination of both. Radiotherapy was administered to 18 patients (8%), 2 of whom had previously undergone surgery. Twelve patients (5.0%) required a second surgical intervention due to incomplete resection of the adenoma (remnants) or recurrence of the disease. No differences were observed in these treatments among the men and women. The median disease duration before definitive treatment was 4 (1–9) years and was not different among the groups. Despite treatment, in 4% (*n* = 9) of the patients, their IGF-1 levels did not normalize. No differences were observed between the men and women.

### 3.2. Echocardiographic Findings

In the echocardiographic parameters of the left and right chambers of the heart, there were no differences in relation to sex. An increase in the ventricular mass index was found in men and women without significant differences. The left ventricular ejection fraction (LVEF) was preserved in 236 patients, with an average of 65.62 ± 6.93%. The left atrium was found to be enlarged in 88 patients, with a left atrial volume index (LAVI) >34 mL/m^2^. LV diastolic dysfunction was found in 26% (*n* = 61) of them, with a mean E wave of 0.76 ± 0.15, a mean A wave of 0.61 ± 0.14, and a mean E/A ratio of 1.30 ± 0.37 (Table 4). Grade I diastolic dysfunction was observed in 17% (*n* = 41) of the patients, grade II was observed in 6% (*n* = 13), and grade III was observed in 3% (*n* = 7).

The most frequent alterations in the left ventricular geometry found were concentric hypertrophy (CHT) in 49% (*n* = 116), cardiac remodeling (CR) in 22% (*n* = 52), and eccentric hypertrophy (EH) in 8% (*n* = 18). The mean right ventricular baseline diameter (RVBD) was 32.69 ± 8.15 mm, and 47 patients (20%) had right ventricular enlargement (RVBD > 40 mm). The right ventricular systolic function (RVSF) was preserved in 99% of the patients, with a mean tricuspid annular plane systolic excursion (TAPSE) of 23.79 ± 2.97 mm and a mean S wave of 13.93 ± 3.99 cm/s. The right atrium was enlarged (a volume greater than 27 mL/m^2^ in women and greater than 32 mL/m^2^ in men) in 51% of the population. Pulmonary hypertension (PASP > 35 mmHg) was found in 28 patients (11.8%), with a mean PASP of 41.67 ± 6.92 mmHg, and the rest had a PASP lower than 35 mmHg (25.28 ± 3.84 mmHg).

Valve involvement was the most frequent finding, occurring in 79% of the study population. Single valve involvement was found in 74 patients (31%), two-valve involvement was found in 85 patients (36%), and in 26 patients (11%), three valves were affected. The most frequent valve disease was tricuspid regurgitation (73%), followed by mitral regurgitation (49%) and aortic regurgitation (24%). Furthermore, 23% (*n* = 55) of the patients had mitral sclerosis, 7% (*n* = 16) had mitral thickening, 4% (*n* = 8) had tricuspid sclerosis, 1% (*n* = 3) had tricuspid thickening, 11% (*n* = 25) had aortic sclerosis, and 5% (*n* = 13) had aortic thickening.

In the total population, the LVEF, LAVI, LVMI, and aortic root diameter were not correlated with the GH or IGF-1 levels or the duration of disease. E wave (ρ = −0.129, *p* = 0.047), A wave (ρ = 0.187, *p* = 0.004), E/A ratio (ρ = −0.173, *p* = 0.008), and E/e′ ratio (ρ = −0.148, *p* = 0.023) were significatively correlated with the duration of disease. None of these factors were correlated with IGF-1 levels. Using the chi-squared test, we observed that elevated IGF-1 was associated with eccentric hypertrophy (*p* = 0.023). However, after adjusting for the presence of obesity and hypertension, this association also lost its significance (*p* = 0.635). Furthermore, elevated IGF-1 levels were not associated with concentric hypertrophy, concentric remodeling, MV insufficiency, AV insufficiency, diastolic dysfunction, right ventricle dilation, right atrium enlargement, pulmonary hypertension, or tricuspid valve insufficiency. Finally, using a logistic regression analysis, we observed whether the presence of obesity, hypertension, diabetes, or a combination of these comorbidities was associated with the echocardiographic alterations observed in our population. Diabetes and diabetes in combination with obesity and hypertension were not associated with any of the cardiac parameters evaluated. Hypertension was associated with right atrium enlargement (*β* = 0.877, *p* = 0.025) and with concentric hypertrophy (*β* = 0.929, *p* = 0.002) but was not associated with any other parameter. Obesity was associated with left atrium enlargement (*β* = 0.726, *p* = 0.014) and concentric hypertrophy (*β* = 0.687, *p* = 0.014) but not with the other findings.

## 4. Discussion

In recent years, the mortality due to cardiovascular causes in patients with acromegaly has decreased [16]. However, some patients have systemic complications at diagnosis, which persist despite adequate GH/IGF-1 control [17]. Therefore, to determine the anatomical and functional cardiovascular alterations in patients with acromegaly, it is necessary to use a non-invasive diagnostic method such as echocardiography on an annual basis [18]. We observed that the most frequent echocardiographic alterations in our study population with acromegaly were left ventricular hypertrophy, left ventricular diastolic dysfunction, left atrial enlargement, right chamber dilatation, and valvular heart disease. It is noteworthy that our population had higher prevalences of T2D, hypertension, obesity, and dyslipidemia, which may have impacted the echocardiographic findings. These diseases were highly prevalent in our population [19]. In a similar way, Hinojosa-Amaya et al. conducted a retrospective study that included 190 patients with acromegaly from an Institutional Review Board database of all patients with a pituitary adenoma from 2006 to 2018. Of these, 46% were controlled, 54.8% had hypertension, and 53.5% had prediabetes or diabetes. Similarly to our study, their patients had an LVEF of 64%, and heart valve disease was present in 87.3%. Meanwhile, their patients had a higher TAPSE (33.1 mm), higher frequencies of concentric hypertrophy (64.7%) and eccentric hypertrophy (35.3%), and a lower frequency of left atrial dilation (26.3%) than these values as reported in our study [20]. In another study by Popielarz-Grygalewicz et al. that evaluated 140 patients with acromegaly, 65% were recently diagnosed and 35% had a long history of the disease; there was a mean age of 51 years and a mean BMI of 30 kg/m^2^; 57% had hypertension, 25% diabetes, and 32% prediabetes; and they described a higher LAVI (41.4 mL/m^2^) and a lower E/A (0.9) but similar values for the LVMI (133.5 g/m^2^), E/e′ (10), and aortic root diameter (33 mm) and pulmonary hypertension (18%) compared with our study’s findings [21].

In patients with acromegaly, GH and IGF-1 concentrations are elevated [22]. The elevation in the GH/IGF-1 axis influences three cardiovascular aspects: myocyte growth, with alterations in their structure; cardiac contractility; and cardiovascular function. The regulation of the myocyte growth process is enhanced by protein synthesis, cardiomyocyte size, and cardiac muscle gene expression. Serum GH/IGF-1 concentrations activate cardiac growth in parallel, causing biventricular hypertrophy [23].

In our study, concentric LV hypertrophy was observed in 57% of the cohort, considered within the range reported in the literature [24]. Petrossians et al. [25] reported a prevalence of left ventricular hypertrophy of 16% in patients with a late diagnosis of acromegaly (6–13 years of evolution) and with an older age at diagnosis. The study population consisted of 3173 patients from 14 acromegaly study centers within the European Union. On the other hand, Lombardi et al. [26] reported a prevalence of 79% in a population of 19 patients in a multicenter study, with most of these patients being at the intermediate stage of the disease.

After the treatment of acromegaly, GH levels decrease, and in turn, hypertrophy decreases. However, in patients in whom high GH and IGF-1 levels persist, this reversal is slower, or the condition is even irreversible [27,28]. The overexpression of IGF-1 increases the transcription of cardiac-muscle-specific genes, including troponin chains, tropomyosin light chains, alpha-actin, and IGF-1 proteins, which cause fibrosis and sarcomerogenesis, causing increased collagen synthesis in the fibroblasts, which causes cardiac collagen deposition, with interstitial remodeling and alterations in LV relaxation and the onset of diastolic dysfunction [29,30].

In our study, we found that 26% of the patients had diastolic dysfunction. Diastolic dysfunction is a common condition in acromegaly, found in 11 to 58% of patients. However, it is usually mild and without clinical consequences [31]. The difference in prevalence observed may be explained by the fact that the patients in our population had undergone transsphenoidal resection of the pituitary adenoma or had already undergone medical treatment, which has been associated with an improvement in diastolic function [32].

The reason for the increased left atrial volume found is unclear, which may have resulted directly from LVH and alterations in diastolic function or increased GH receptor expression in the atrial cardiomyocytes [21]. In our study, 37% of the patients had left atrial dilatation. Ilter et al. compared the LA volume in patients with acromegaly with that in healthy patients of a similar age and sex, with no significant difference found between groups and no relation with their serum levels of GH or IGF-1 [33]. The presence of left atrial enlargement induces the presence of arrhythmias and is cause for performing 24 h Holter monitoring in these patients [21,34].

Within the study population, 47 patients showed RV dilatation. Acromegaly has been associated with increased free wall thickness, RV dilatation, and diastolic dysfunction [35,36] and decreased ventricular function [37]. However, there is scarce information on RV involvement in acromegaly [38].

Pulmonary arterial hypertension (PAH) may be directly associated with acromegaly [39], and IGF-1 has been shown to remodel the bronchial vascular network in animals [40,41]. Nevertheless, it is a disorder that can be found in different clinical entities and can complicate cardiovascular and respiratory diseases [42]. In addition, it is associated with other complications of acromegaly, such as obstructive sleep apnea, SAH, and left and right ventricular hypertrophy [43,44]. Therefore, we propose that more studies be performed to verify whether there is a direct relationship between acromegaly and PAH.

Valvular involvement was the most frequent finding in this population, occurring in 78% of patients. This is similar to the figures reported in other studies [20,45]. In our research, the most frequently affected valve was the tricuspid valve, while other studies have reported a higher prevalence of aortic and mitral valve disease [1,46]. Acromegaly is associated with an increased prevalence of valvular heart failure. An increase of up to 19% in the probability of developing valvular regurgitation has been reported in patients with acromegaly [7,46]. This has been related to the duration of their exposure to high concentrations of GH [45]. Due to all of the characteristics mentioned above, echocardiographic follow-up is essential in these patients [45]. The high prevalence of valvulopathy in acromegaly can be explained by its late diagnosis, the long evolution of the disease, having active disease, and continued exposure to high concentrations of GH and IGF-1 [9,10]. However, Giustina et al. reported that valvular heart disease might not be related to acromegaly since tricuspid insufficiency is a common condition in the general population and may be associated with other factors, such as hypertension [3].

The limitations of our study include it having been conducted at a tertiary-level concentration hospital, so its findings cannot be generalized. However, this is the center of the greatest reference for patients with acromegaly in Mexico. Another area for improvement is that this was a retrospective and cross-sectional study, so there was no continuous follow-up of the patients. However, this study is a precedent for the generation of new hypotheses, and because these patients are being followed up, prospective studies could be performed in the future.

## 5. Conclusions

Although acromegaly is a disease of low prevalence, cardiovascular diseases remain as relevant causes of morbidity and mortality. Echocardiography is the primary non-invasive method for detecting the main cardiac changes in and optimizing the follow-up of these patients. The main cardiac modifications demonstrated in our study were changes in LV morphology, LA morphology, diastolic function, RV, RA, and valvular abnormalities.

## Figures and Tables

**Table 1:** IGF-1 levels according to sex and age.

Age	Women (ng/mL)	Men (ng/mL)
18–30 years	151–353	172–432
31–40 years	116–249	122–304
41–50 years	84–191	87–190
51–60 years	54–177	54–177
>61 years	56–140	56–140

**Table 2:** Clinical parameters and comorbidities of the studied population.

Clinical Parameters	Men (*n* = 89)	Women (*n* = 148)	*p* *
Age, years	48 ± 12	49 ± 13	0.223
Height, m	1.69 ± 0.09	1.57 ± 0.07	0.146
Weight, kg	83 (77–93)	72 (64–83)	<0.001
BMI, kg/m^2^	29.2 (26.7–32.2)	29.4 (25.5–33.9)	0.938
Obesity, *n* (%)	34 (14)	72 (30)	0.013
Smoking, *n* (%)	9 (4)	7 (3)	0.033
Diabetes Mellitus, *n* (%)	25 (11)	60 (25)	0.982
Prediabetes, *n* (%)	16 (7)	32 (13)	0.745
Hypertension, *n* (%)	25 (11)	57 (24)	0.734
Dyslipidemia, *n* (%)	11 (5)	21 (9)	0.690
Secondary Hypogonadism, *n* (%)	13 (5)	4 (2)	0.001
Hypocortisolism, *n* (%)	3 (1)	8 (3)	0.471
Hypothyroidism, *n* (%)	21 (9)	62 (26)	0.028

BMI = body mass index. The results are reported as the means ± standard deviations or *n* (%). * Evaluated using the chi-squared test, Student’s *t*-test, or the Mann–Whitney U test.

**Table 3:** Biochemical values of the studied population.

Biochemical Values	Men (*n* = 89)	Women (*n* = 148)	*p* *
Hemoglobin, mg/dL	15.2 (14.1–16.0)	13.7 (12.5–14.3)	<0.001
Hematocrit, %	44.8 (42.7–47.3)	41.1 (37.9–43.2)	<0.001
Glucose, mg/dL	98 (90–105)	103 (93–111)	0.020
Urea, mg/dL	31 (25–38)	28 (23–37)	0.050
Creatinine, mg/dL	0.85 (0.77–0.94)	0.67 (0.58–0.77)	<0.001
Cholesterol, mg/dL	171 (150–199)	188 (164–212)	0.006
HDL, mg/dL	46 (39–56)	52 (40–63)	0.018
LDL, mg/dL	92 (74–112)	99 (79–122)	0.156
Triglycerides, mg/dL	131 (104–170)	147 (114–209)	0.025
BUN, mg/dL	14.1 (11.8–18.0)	13.1 (10.7–17.2)	0.055
Hb1Ac, %	5.7 (5.5–6.3)	6.0 (5.5–6.6)	0.076
Prolactin, ng/ml	8.6 (2.9–15.2)	7.3 (1.0–13.6)	0.352
Free T4, ng/dL	1.23 (1.08–1.37)	1.23 (1.10–1.40)	0.797
TSH, μUI/ml	2.0 (1.04–2.62)	1.38 (0.52–2.79)	0.063
GH, ng/ml	0.94 (0.35–2.89)	1.22 (0.65–2.65)	0.132
IGF-1, ng/ml	250 (181–405)	240 (170–334)	0.114

HDL: high-density lipoprotein; LDL: low-density lipoprotein; BUN: blood urea nitrogen; Hb1Ac: glycated hemoglobin; Free T4: free thyroxine; TSH: thyroid-stimulating hormone; GH: growth hormone; IGF-1: insulin-like growth factor type 1. The results are reported as means ± standard deviations. * Evaluated using Student’s *t*-test or the Mann–Whitney U test.

**Table 4:** Echocardiographic changes in the left and right chambers of the heart in the studied population.

Parameter	Men (*n* = 89)	Women (*n* = 148)	*p* Value *
Concentric Hypertrophy, *n* (%)	40 (19)	76 (32)	0.339
Eccentric Hypertrophy, *n* (%)	6 (3)	12 (5)	0.701
Concentric Remodeling, *n* (%)	24 (10)	28 (12)	0.147
Normal Geometry, *n* (%)	19 (8)	32 (14)	0.960
LAVI, mL/m^2^	28.21 ± 9.97	26.88 ± 8.59	0.058
LVMI, g/m^2^	115.64 ± 43.04	115.269 ± 45.26	0.227
Aortic Root, mm	31.51 ± 4.69	28.06 ± 3.83	0.003
LVEF, %	64.68 ± 7.67	66.18 ± 6.4	0.562
MV Insufficiency, *n* (%)	40 (17)	73 (31)	0.513
AV Insufficiency, *n* (%)	9 (4)	12 (5)	0.599
TV insufficiency, *n* (%)	66 (28)	98 (41)	0.200
Diastolic Dysfunction, *n* (%)	27 (11)	34 (14)	0.209
E, m/s	0.71 ± 0.17	0.78 ± 0.12	0.000
A, m/s	0.58 ± 0.12	0.62 ± 0.15	0.014
E/A	1.27 ± 0.37	1.32 ± 0.38	0.971
e′, cm/s	8.43 ± 2.95	8.8 ± 3.38	0.732
E/e′	9.27 ± 5.1	9.14 ± 3.42	0.735
Right Ventricle Dilation, *n* (%)	20 (8)	21 (9)	0.103
Right Atrium Enlargement, *n* (%)	16 (7)	19 (8)	0.280
Pulmonary Hypertension, *n* (%)	7 (3)	21 (9)	0.139

LAVI: left atrial volume index; LVMI: left ventricular mass index; LVEF: left ventricular ejection fraction; MV: mitral valve; AV: aortic valve; TV: tricuspid valve; E: E wave; A: A wave; E/A: E wave and A wave ratio; e′: mitral annular velocity; E/e′: E/e′ ratio. The results are reported as means ± standard deviations or *n* (%). * Evaluated using the chi-squared test or Student’s *t*-test.

## Data Availability

The data analyzed in this study are available from the corresponding author upon reasonable request.

## Abbreviations

The following abbreviations are used in this manuscript:

CHT	Concentric hypertrophy
CR	Concentric remodeling
EH	Eccentric hypertrophy
LVEF	Left ventricular ejection fraction
LAE	Left atrial enlargement
VI	Valvular insufficiency
GH	Growth hormone
IGF-1	Insulin-like growth factor type 1
LVH	Left ventricular hypertrophy
LV	Left ventricular
PSAP	Pulmonary artery systolic pressure
HbA1c	Glycated hemoglobin
TSH	Thyroid-stimulating hormone
LAVI	Left atrial volume index
RVBD	Right ventricular baseline diameter
RVSF	Right ventricular systolic function
TAPSE	Tricuspid annular plane systolic excursion
